# Developing Emotional Design: Emotions as Cognitive Processes and their Role in the Design of Interactive Technologies

**DOI:** 10.3389/fpsyg.2017.01773

**Published:** 2017-10-09

**Authors:** Stefano Triberti, Alice Chirico, Gemma La Rocca, Giuseppe Riva

**Affiliations:** ^1^Department of Psychology, Università Cattolica del Sacro Cuore, Milan, Italy; ^2^Independent Researcher, Milan, Italy; ^3^Applied Technology for Neuro-Psychology Laboratory, Istituto Auxologico Italiano, Milan, Italy

**Keywords:** emotional design, user centered design, emotions, user experience, appraisal, complex emotions

In the last 20 years, the debate on the role of *emotions* in the field of industrial design has grown exponentially. Emotional Design emerged as the effort to promote positive emotions (Norman, 2007) or pleasure in users (Jordan, 2002; Green and Jordan, 2003) by means of design properties of products and services. According to Van Gorp and Adams (2012), design based on emotions can affect overall user experience deeply, since emotions influence decision making, affect attention, memory, and generate meaning. It is possible to identify two main approaches to applied emotional design. The first is based on the modification of object's aesthetic appearance or interface, the latter focuses on promoting fluent and engaging interactions.

Both these approaches pertain to technology design, which includes especially common-use technological products. Regarding the first approach, several studies showed the importance of emotional aspects as drivers of market success, enjoinment, and active usage of technologies. For instance, Desmet et al. (2007) demonstrated that users attributed a “wow effect” (i.e., the combination of fascination, pleasant surprise, and desire) to those cellphones having some pleasant features in their exteriors. Studies in multimedia learning (Um et al., 2012; Plass et al., 2013) showed that embedding emotional stimuli (e.g., face-like shapes, vibrant colors) into interfaces elicited positive emotions in learners and improved learning outcomes.

The second perspective considers fluid interactions as a fundamental factor for an overall positive experience of use (Hancock et al., 2005; Hassenzahl and Tractinsky, 2006). This approach includes design based on the concept of psychological flow, namely an optimal experience of total absorption in a task when agent's skills and environmental challenges are both at a high level and balanced (Csikzentmihalyi, 1988; Csikszentmihalyi, 2002). Research demonstrated that flow experience is quite common in technology usage (Pilke, 2004; Triberti et al., 2016), such as in video games (Cowley et al., 2008; Jin, 2012; Argenton et al., 2014) and personal computer-mediated activities (Voiskounsky and Smyslova, 2003; Skadberg and Kimmel, 2004). For this reason, flow-inspired design models have been created and applied to the design of interactive digital technologies such as educational games and augmented reality (Alexiou et al., 2012; Neal, 2012). Other approaches for promoting emotions by engagement are gamification or the inclusion of game mechanics in interfaces (such as, prizes, achievements…) and interactive storytelling, which frames interaction within emotional scenarios with compelling characters, events, and motives (Morford et al., 2014).

The objective of the present contribution is to extend the discourse on emotional design, highlighting that technology designers can rely on other components beyond the above-mentioned aesthetic and engagement ones, in order to create innovative and effective devices. Indeed, emotions have further aspects that could be exploited by emotional designers. For instance, emotions are also cognitive processes—based on appraisal component—with a notable influence on the overall quality of interaction. According to this perspective, new technologies can be considered and treated as opportunities to manipulate, enhance and trigger different discrete, and even complex emotional states. Finally, emotions can “participate” to interactions (instead of being a mere byproduct of it), by providing inputs to digital technologies to modify or influence final outputs.

This contribution explores opportunities provided by conceiving emotions as cognitive processes and active agents of interactions, in the field of emotional design.

Since *Affective computing* studies (Picard, 2003; Tao and Tan, 2005), designer have developed computers able to sense, recognize, and express emotions. New technologies combined with ubiquitous and wearable sensing become able to adapt to users' actual emotional states. For example, video games content changes (e.g., becoming more or less challenging) according to gamers' emotional state (e.g., bored or frustrated; Gilleade et al., 2005). Also mobile apps have been integrated with biofeedback sensors to promote positive emotions and relaxation (Serino et al., 2014). For instance, users can learn to monitor and control their emotional states by looking at virtual environments features (e.g., a burning fire) changing according to their psychophysiological activation. *Affective Design* (Reynolds and Picard, 2001) has shown that “emotional design” could be conceived not only as the inclusion of pleasant and/or engaging aspects in interfaces to augment pleasure, but also as the recognition and measurement of emotions to provide inputs to the technology and modify its functioning.

However, we argue that this approach, which is mainly based on general affect and moods, can be extended to discrete emotions, each characterized by a specific pattern of appraisal (i.e., emotion's cognitive profile). Studies on appraisal showed that an emotional episode emerges when one evaluates his/her own relationship with the surroundings (Roseman, 1991; Smith and Lazarus, 1993; Aue and Scherer, 2008; So et al., 2015). This automatic and subjective evaluation is based on specific properties of the stimulus such as relevance and congruence to personal goals or agency (oneself, others, or impersonal causes of the event), coping potential and control (Moors et al., 2013). The results of such evaluations bring about specific discrete emotions. Discrete emotional events are separable, distinguishable, and identifiable emotional state inducing changes into psychophysiology, behavior, motivation, judgment, and experience (Lench et al., 2011). Specifically, a discrete emotional event such as surprise, disgust, fear, would emerge after this first evaluation of the stimulus. After the appraisal component has been activated, a motivation to approach or avoid the stimulus follows (Moors et al., 2013). Furthermore, also changes in physiological parameters are involved, ranging from perspiration to muscle contraction. Finally, emotions are subjectively felt, since they can be described by the subject or can be quantified through numerical scales (Harmon-Jones et al., 2016), usually based on arousal (high/low intensity) and valence (positive/negative) aspects of the emotion at least (Mattek et al., 2017).

In our opinion, the scientific knowledge of discrete emotions based on their cognitive components—*appraisal—*can be easily translated into initial guidelines to develop a cognitive science-informed emotional design.

For instance, a field in which a partial discrete emotional approach was combined with affective is automotive technologies design (Ho and Spence, 2013). Nasoz et al. (2010) successfully tested a multi-modal intelligent car interface based on psychophysiological signals, able to classify driver's discrete emotional state as fear, boredom or anger that can be used to tune multisensory features of the car environment accordingly to help prevent accidents. In this case, technologies provide unprecedented opportunities to record even discrete users' emotional states (**monitoring emotions**), in order to tailor final outcomes. Future research in emotional design may explore how the continuous measurement of specific emotions can be exploited to influence ongoing interaction with common-use technology, for example modifying real-time easiness of use of devices or selecting digital content depending on the users' ongoing emotional responses.

A lot have been done, but we argue that still more can be done relying on an appraisal-based discrete emotion design approach. Indeed, appraisal theories of emotion have a lot to offer emotional design (Desmet, 2003; Bordegoni et al., 2014; Oatley and Johnson-Laird, 2014). Drawing on the scientific literature on discrete emotions as cognitive process, it is possible to *expand the kinds of emotions* that designers can reproduce and promote. Insofar emotions are considered as discrete events emerging from a specific pattern of appraisal themes (Smith and Lazarus, 1993), the more these themes are detailed, the higher the number of emotions and *emotional nuances* a designer can detect and control. For instance, sadness' core appraisal concerns an irrevocable loss (Smith and Ellsworth, 1985; Lazarus, 1991). If we detail this core appraisal, we can distinguish different kinds of sadness, such as melancholy, disappointment.

Such approach not only allows distinguishing different emotional nuances but it can also provide suggestions about reaching and promoting specific *complex* emotional states which include several single discrete emotional sub-components. Indeed, intervening on aesthetic appeal of interfaces allows designers to promote a general positive feeling in users, that is what has been done by most current approaches. However, the scientific literature can provide indications to elicit even specific complex emotions simply basing on their pattern of appraisal. For instance, one is the emotion of *awe* or the deep feeling of wonder, astonishment and fear people experience when facing stimuli perceived as incredible and incommensurable (Keltner and Haidt, 2003) (e.g., looking at vast panoramas; witnessing childbirth; etc.). Emotional appraisal leading to the experience of awe includes two distinctive elements, namely the feeling of vastness (perceptual or conceptual) and need for accommodation (i.e., the need for updating one's mental schemas to adapt them to the extraordinary). Recent research demonstrated that immersive technologies (e.g., Virtual Reality and 360° immersive videos) can be used to induce profound awe experiences in controlled environments, such as the lab (Gallagher et al., 2014; Chirico et al., 2016, 2017; Gaggioli et al., 2016). For instance, Chirico et al. (2017) were able to grasp subtle differences in the emergence of awe considering both self-reported and psychophysiological measures of this emotion. Awe resulted in a “freezing” response in front of something perceived vast and whose intensity can be enhanced by placing a user inside a 360° immersive virtual environment even with a low degree of interactivity. Appraisal dimensions of this emotion were analyzed in relation with the psychophysiological ones, thus providing a clearer picture of the emotional process.

In the emotional design, another important aspect concerns that emotions are closely intertwined over a continuous stream within subjects' experience. The sub-components of emotional episodes influence each other and subsequent emotional responses. For example, sad people are more likely to attribute agency of subsequent stimuli to others and the external world, because sadness is an emotion experienced toward events one cannot control (Han et al., 2007). Angry people are more likely to transfer anger to the next event to be evaluated in the surroundings (Beaudry et al., 2010; Darban and Polites, 2016).

In other words, emotions do not appear “out of nowhere” as the simple byproduct of a given stimulus and its appraisal. Instead, they are influenced by previous emotional states, or pre-existing individual traits, dispositions, and contextual factors (Verduyn and Brans, 2012; Kim et al., 2016). Therefore, a technology designer working with emotions should be able to identify and measure *emotional profiles* or pre-existing individual/contextual characteristics that can influence the effectiveness of emotion-based technological services. For example, smartphones can be designed to elicit reactions such as surprise (Desmet et al., 2007). Nevertheless, such emotional state is not lasting in time, rather it tends to disappear shortly after the first encounters with the stimulus, since surprise arises from unexpected and novel events (Horstmann, 2006). Emotional designer should be able to create technologies updating according to users' personal information, in order to renovate the emotion of surprise continuously. In other words, they should design technological products able to actively adapt their outcomes to users' everyday life in line with individuals' peculiarities. This would allow designers promoting lasting emotional benefits such as loyalty, satisfaction, and possibly happiness and well-being. Although such ability largely depends on the designer's ability, it is possible to empower one's capacity to analyze emotional profiles of users by employing User Centered Design research techniques (Abras et al., 2004; Garrett, 2010; Lowdermilk, 2013; Triberti and Liberati, 2014; Triberti and Barello, 2016), especially those involving the observation of users in the context of use (Viitanen, 2011) and those resuming typical users' needs and emotional benefits (Osterwalder and Pigneur, 2010; Miaskiewicz and Kozar, 2011). Collecting data on users' habits, intentions and context could help the designer to tailor technologies on their pre-existing emotional stream, within a user-centered design framework.

Finally, the advancement of common-use technology, combined with the knowledge available in cognitive science literature, could provide designers with extraordinary possibilities to fully exploit emotions' potential for user experience (see Figure 1 for resume). In our opinion, this new approach could be based on: (1) the assessment of discrete emotions in an ongoing interaction to provide on-line modifications of interfaces (affective computing/affective design); (2) relying on scientific literature on emotions as discrete cognitive processes, to promote even complex emotions, and (3) analyzing users' “emotional profiles” to tailor technologies on their pre-existing emotional traits, within a user-centered design framework.

**Figure 1:**
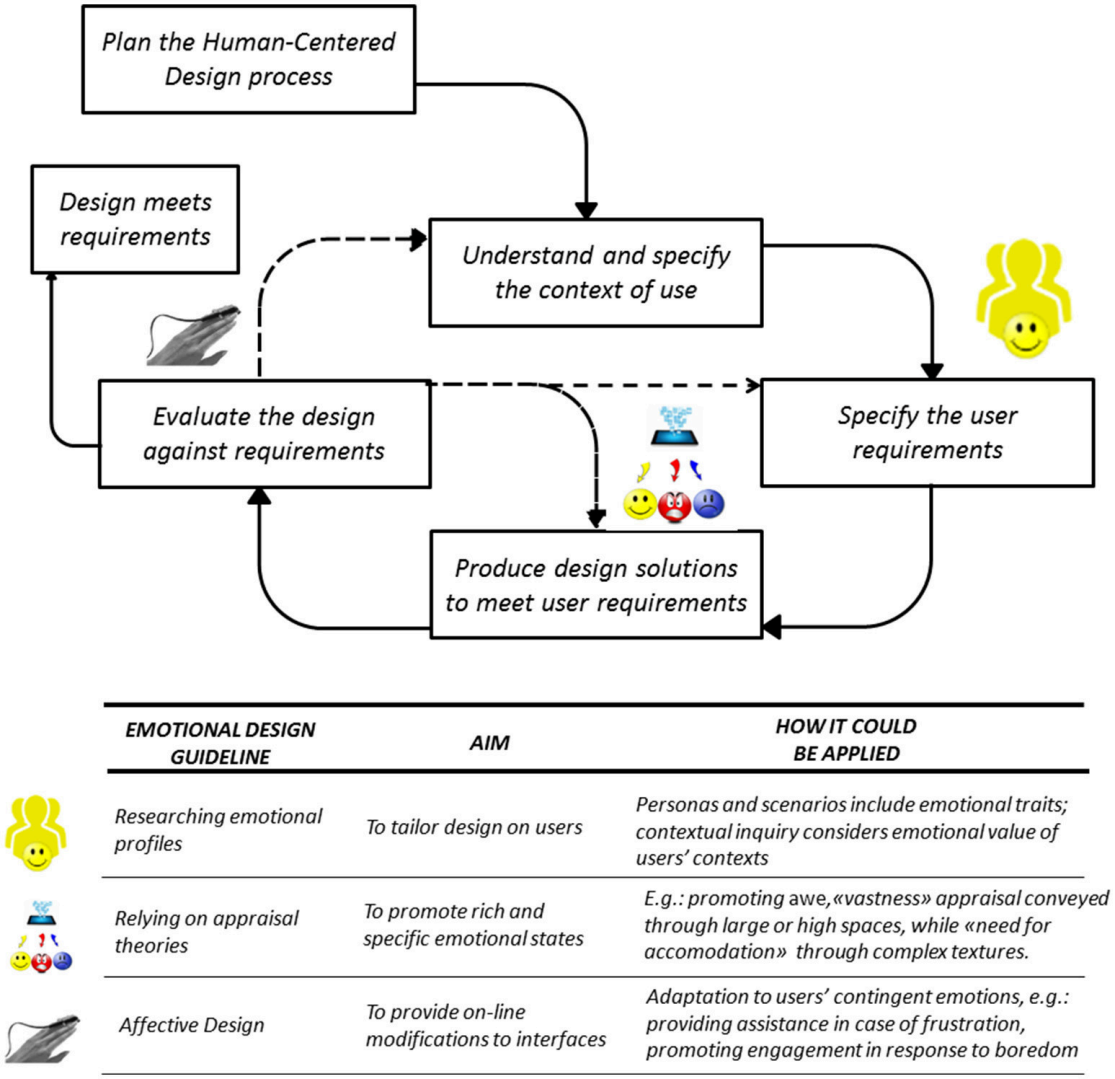
A resume of the development guidelines for a “scientific” Emotional Design, based on the human centered design phases according to ISO 9241 (hatching stands for possible iteration). While the second guideline in the table regards appraisal-based generation of emotion, the first and the third constitute examples of emotions participating in design.

## Author contributions

ST conceived the ideas presented in the article and wrote the first draft. AC assisted in drafting the manuscript and contributed with important intellectual content. GLR edited the manuscript from a design perspective and created the image. GR supervised the whole process and contributed with important intellectual content.

### Conflict of interest statement

The authors declare that the research was conducted in the absence of any commercial or financial relationships that could be construed as a potential conflict of interest.
